# Recovery from chronic PFAS exposure can reverse chemotherapy resistance and mitochondrial alterations in ovarian cancer cells

**DOI:** 10.1016/j.toxlet.2026.111858

**Published:** 2026-02-19

**Authors:** Brittany P. Rickard, Lauren A. Sapienza-Lundie, Vesna A. Chappell, Suzanne E. Fenton, Imran Rizvi

**Affiliations:** a Department of Biological Sciences, North Carolina State University, Raleigh, NC 27606, USA; b Curriculum in Toxicology & Environmental Medicine, University of North Carolina School of Medicine, University of North Carolina at Chapel Hill, Chapel Hill, NC 27599, USA; c Lampe Joint Department of Biomedical Engineering, University of North Carolina at Chapel Hill, Chapel Hill, NC 27599, USA, and North Carolina State University, Raleigh, NC 27606, USA; d Mechanistic Toxicology Branch, Division of Translational Toxicology, National Institute of Environmental Health Sciences, Research Triangle Park, NC 27709, USA; e Center for Human Health and the Environment, North Carolina State University, Raleigh, NC 27606, USA; f Lineberger Comprehensive Cancer Center, University of North Carolina School of Medicine, Chapel Hill, NC 27599, USA; g Center for Environmental Health and Susceptibility, University of North Carolina at Chapel Hill, Chapel Hill, NC 27599, USA

**Keywords:** Perfluoroalkyl substances, PFAS, Recovery, Ovarian cancer, Environmental exposures, Chemoresistance, Mitochondria

## Abstract

Per- and polyfluoroalkyl substances (PFAS) are environmental contaminants of global concern that have been associated with a variety of adverse health outcomes, including diminished chemotherapy response. Previous studies in moderately chemosensitive ovarian cancer cells (OVCAR-3) have shown that the induction of chemoresistance from PFAS exposure is duration-dependent, with longer, more human-relevant exposure durations leading to worse outcomes. Mitochondrial content was also altered following chronic PFAS exposure, suggesting mitochondria as contributors to PFAS-induced chemoresistance. Here, chemotherapy response following chronic PFAS exposure in a chemoresistant human ovarian cancer cell line, OVCAR-8, was evaluated. Compared to OVCAR-3 cells, chemotherapy response was unaffected by chronic PFAS exposure in OVCAR-8 cells. As individuals gain awareness of sources of PFAS exposure, and associated harmful effects, actions can be taken to limit exposure using water filtration systems and/or safer alternatives to PFAS-containing consumer goods. Thus, we also explored the ability of PFAS-sensitive OVCAR-3 cells to recover from chronic exposure. Following 6 passages of chronic PFAS exposure, cells were “outgrown” in the absence of PFAS for 7 additional passages and proliferation, chemotherapy response, and mitochondria-related alterations were assessed. Compared to chronically-exposed cells, outgrown cells displayed heightened sensitivity to chemotherapy along with decreased superoxide production and mitochondrial content. Proliferation remained significantly elevated compared to controls, suggesting that not all PFAS-induced effects are abrogated by a recovery period. Together, these findings suggest that ovarian cancer cells differ in their PFAS sensitivities and that mitochondria-related alterations resulting from chronic PFAS exposure can be reversed following a “recovery period”, potentially resensitizing cancer cells to chemotherapy.

## 1. Introduction

Exposures to environmental contaminants, such as per- and polyfluoroalkyl substances (PFAS), are ubiquitous in daily life (Sunderland et al., 2019). PFAS are a class of man-made chemicals, many of which are resistant to degradation, leading to their environmental and bodily bioaccumulation upon exposure (Buck et al., 2011). A main route of PFAS exposure is contaminated drinking water supplies, although other routes include personal care products and food-associated sources (Sunderland et al., 2019). Since humans are likely exposed to PFAS daily through multiple routes, it is critical to consider not only how long-term exposure affects disease onset, progression, and treatment outcomes, but also steps that can be taken to limit exposures.

The majority of studies investigating the adverse health effects of PFAS in cell culture-based systems focus on short-term (ST) exposures. For instance, studies evaluating the effects of PFAS on breast, placenta, and ovarian cells have examined 24, 96 hour (h) exposures (Blake et al., 2022; Clark et al., 2024; Liu et al., 2024; Pierozan et al., 2023; Rickard et al., 2022), while those in thyroid and ovarian cancer cells have used 6 day (d) exposure durations (Coperchini et al., 2021; Rickard et al., 2025). Although ST exposure studies are critical to understand the effects of, and mechanisms by which, PFAS target varying cell types, there is an unmet need for chronic PFAS exposure studies to gain a better understanding of adverse impacts associated with human-relevant exposure durations.

Our recent study was one of the first to evaluate the effects of chronic, human-relevant concentrations of an emerging PFAS exposure in a monolayer culture model and the first to do so in an ovarian cancer cell line (OVCAR-3) (Rickard et al., 2025; Qi et al., 2023). We reported that chronic (~35 d) PFAS exposures induce chemoresistance in a duration-dependent manner compared to 48 h and 6 d exposures. We also proposed that PFAS target mitochondria, in part, to induce chemoresistance, as evidenced by altered bioenergetics, mitochondrial reactive oxygen species (ROS) production, and mitochondrial content.

Since OVCAR-3 cells are moderately chemosensitive, the first goal of this study was to evaluate the impacts of chronic PFAS exposure in a more aggressive and chemoresistant cell line: OVCAR-8 (Bicaku et al., 2012; Hallas-Potts et al., 2019). On a molecular level, these cell lines differ in their mutational profiles, with OVCAR-3 cells overexpressing CCNE1 and C11orf30 and OVCAR-8 cells harboring mutations in ERBB2, KRAS, and CTNNB1 (Domcke et al., 2013). Additionally, OVCAR-8 cells have lower oxidative phosphorylation subunit expression, higher reactive oxygen species levels, and decreased levels of antioxidant scavenging enzymes than OVCAR-3 cells, all of which may correlate with the enhanced innate invasiveness and reduced chemosensitivity of this cell line (Koc et al., 2023). Comparing the effects of chronic PFAS exposure in two molecularly and bioenergetically distinct cell lines may help inform mechanisms driving chemoresistance.

Since PFAS contamination is a global concern, and those living in contaminated communities are gaining a better understanding of their adverse effects, many individuals take action to limit their overall exposure. Such actions can include installing water filtration systems (Herkert et al., 2020), purchasing PFAS-free consumer goods, and, in some cases, moving geographical locations, as these are known determinants of PFAS exposure (Park et al., 2019). Thus, another goal of this study was to examine how recovery (or outgrowth) of PFAS chronically-exposed ovarian cancer cells, defined as seven passages without PFAS exposure, affects proliferation, chemotherapy response, and mitochondrial parameters (mitochondrial membrane potential (ΔΨ_m_), ROS production, content, and mitochondrial DNA (mtDNA) copy number). This recovery or “wash-out” period is an attempt to reduce cellular PFAS levels, and mimic what may occur in this cell type if individuals make conscious attempts to reduce PFAS exposures over time. Importantly, recovery was only examined in cell lines where chemoresistance following chronic PFAS exposure was observed. We also explored how re-exposure of outgrown cells to PFAS, which could mimic short-term travel to a contaminated area or unbeknownst consumer exposures, affects these endpoints. Together, these findings could inform cellular adaptations contributing to PFAS-induced chemoresistance and the ability of cells to revert to normal functionality following chronic PFAS exposures.

## 2. Methods

### 2.1. Cell culture

Human ovarian adenocarcinoma OVCAR-3 cells were obtained from the American Type Culture Collection (Manassas, VA, USA) and OVCAR-8 cells were obtained from the University of Maryland. OVCAR-3 cells were grown in RPMI 1640 (Cytiva HyClone^™^, Marlborough, MA, USA) supplemented with 20 % fetal bovine serum (FBS, Cytiva HyClone^™^), 100 U/mL penicillin and 100 μg/mL streptomycin (Sigma-Aldrich, St. Louis, MO, USA), and 0.1 mg/mL human recombinant insulin solution (Gibco, Billings, MT, USA). OVCAR-8 cells were grown in RPMI 1640 supplemented with 10 % FBS, 100 U/mL penicillin and 100 μg/mL streptomycin. Cells were maintained in monolayers at 37°C in a humidified incubator with 5 % CO_2_ and regularly tested for mycoplasma contamination.

### 2.2. PFAS exposures

PFAS stocks were prepared at 10 mM in 1 M potassium hydroxide in methanol (Lab Chem Inc., Zelienople, PA, USA): perfluorooctanoic acid (PFOA, CAS#335–67–1, Synquest Laboratories, Alachua, FL, USA), perfluoroheptanoic acid (PFHpA, CAS#375–85–9, Sigma-Aldrich), and perfluoropentanoic acid (PFPA, CAS#2706–90–3, TCI America, Portland, OR, USA). Solutions containing basic methanol only are referred to as “vehicle”. PFAS stocks were prepared in glass vials and stored at −20°C. Dosing solutions were supplemented with 25 mM 2-[4-(2-hydroxyethyl)piperazin-1-yl]ethanesulfonic acid acid (HEPES, Gibco) to maintain pH, which was determined at baseline and following the addition of methanol/PFAS.

### 2.3. Development of PFAS chronically-exposed cell lines

OVCAR-3 and OVCAR-8 cells were exposed to 0.5 μM PFHpA for 26–35 days. Briefly, cells were thawed and grown to confluence prior to being split into three flasks. These flasks contained an unexposed control (complete medium), a vehicle chronically-exposed control (complete medium + 1 % vehicle + HEPES), and PFAS chronically-exposed cells (complete medium + 0.5 μM PFHpA in 1 % vehicle + HEPES). Flasks were split every 3–5 days. Experiments using chronically-exposed cells occurred following passage 6. In some cases, stocks of previously grown “chronics” were used for experiments. When using frozen stocks, cells were allowed to grow in complete medium to confluence before being split and re-exposed to the same condition (complete medium, vehicle, or PFAS) as before. Exposure continued until passage 6 was reached.

### 2.4. Development of an outgrown PFAS chronically-exposed cell line

To evaluate the ability of OVCAR-3 cells to recover from chronic PFAS exposure, chronically-exposed cell lines (control, vehicle, and PFAS) were grown for 7 passages in complete medium. Outgrown cells were split every 3–5 days. Once passage 13 was reached (6 chronic exposures + 7 outgrown passages), experiments were performed and the remainder of cells, if any, were frozen. To verify findings, several different batches were developed and/or thawed and used for experiments.

#### 2.4.1. Re-exposure of an outgrown cell line

Several experiments sought to understand how re-exposure of an outgrown cell line affects mechanistic and treatment response outcomes. Cells were seeded at 8.3 * 10^3^ cells/cm^2^ in white-walled, clear bottom 96-well plates, based on the linear dynamic range of the CellTiter-Glo assay (Promega, Madison, WI, USA) (Rickard et al., 2025). One-day post-seeding, cells were exposed to 1 % vehicle, 0.5 µM PFHpA, 2 µM PFHpA, 1 µM PFHpA + 1 µM PFPA (referred to as PFHpA + PFPA), or 0.75 µM PFOA + 0.75 µM PFHpA + 0.75 µM PFPA (referred to as PFOA + PFHpA + PFPA) in serum-free medium for 1 h. 2X serum solutions were then added directly to wells containing serum-free medium for 71 h, so the medium had a normal level of serum for the remainder of exposure. After 72 h, this exposure was repeated for an additional 72 h (including another serum-free pulse) prior to endpoint assays on day 8.

### 2.5. Evaluation of cell survival pre- and post-chemotherapy treatment

Cells were seeded and allowed to grow prior to receiving either complete medium or re-exposure to PFAS as described above. After 72 h, medium was removed and replaced with the same condition concurrent with chemotherapy for 72 h. Carboplatin (TCI America) stocks were prepared (5 mM) in complete medium on the day of dosing, which were used to prepare working solutions (25-800 μM). Doxorubicin hydrochloride (Sigma-Aldrich) stocks (10 mM) were prepared in dimethyl sulfoxide (DMSO) on the day of dosing and used to prepare working solutions (0.08-10 μM). Since exposures were concurrent, PFAS and chemotherapy doses were double the desired concentration (e.g. 4 μM PFHpA + 800 μM carboplatin = 2 μM PFHpA + 400 μM carboplatin final concentration in medium). After 72 h, the CellTiter-Glo Assay was performed.

### 2.6. Evaluation of ΔΨ_m_, superoxide, and mitochondrial content

ΔΨ_m_ was evaluated using two different dye-based methods: 5,5’,6,6’-tetrachloro-1,1’3,3’-tetraethylbenzimidazolocarbo-cyanine iodide (JC-1) and tetramethylrhodamine ethyl ester (TMRE). For JC-1, outgrown OVCAR-3 cells ± re-exposure or secondarily exposed PFAS chronically-exposed cells were seeded at 1.3 * 10^5^ cells/well in blackwalled, clear bottom 96-well plates. Cells were grown for 24 h prior to treatment with JC-1 dye (Invitrogen^™^, Waltham, MA, USA) for 15 min. Dye was removed and cells were washed with phosphate buffered saline (PBS) before being dosed with complete medium (controls) or PFAS + chemotherapy in serum-free medium at twice the final working concentrations following the dosing scheme outlined above. Serum-free medium was used to ensure adequate uptake of PFAS and/or chemotherapy. After 1 h, the JC-1 red:green aggregate ratio was read using the SpectraMax iD3 fluorescence (Molecular Devices, San Jose, CA, USA) or the CLARIOstar Plus microplate reader (BMG Labtech, Ortenberg, Germany). Parameters for measurement: green aggregate—excitation: 488 nm, emission: 529 nm; red aggregate—excitation: 488 nm, emission: 590 nm.

#### 2.6.1. ΔΨ_m_ quantification via flow cytometry

Cells were co-stained with TMRE and MitoTracker^™^ Green FM dyes (Invitrogen^™^). TMRE was used as a proxy of ΔΨ_m_ while MitoTracker^™^ Green was used for normalization of mitochondrial content. Outgrown and re-exposed OVCAR-3 cells were seeded at 1.6 * 10^4^ cells/cm^2^ in 12-well plates (Rickard et al., 2025). After 24 h, medium was removed and cells received either complete medium or PFAS exposure via the dosing scheme outlined above. On day 8, medium was removed, and cells were washed with PBS before trypsinization. Complete medium was then added to deactivate trypsin and cell suspensions were transferred to a microcentrifuge tube before being counted using the Countess 3 FL Automated Cell Counter (Thermo Fisher) and centrifuged at 1 * 10^3^ rpm for 5 min. Supernatant was removed and cells were resuspended at 5 * 10^5^ cells/mL. Resuspensions were transferred to flow cytometry tubes and co-stained with 2.5 μL of a 5 μM TMRE stock (final = 25 nM) and 2.5 μL of a 1 mM MitoTracker^™^ Green FM stock (final = 50 nM) for 15 min at 37°C. TMRE was read using the PE-Texas Red Channel (Excitation: 561 nm; Emission: 610 nm) and MitoTracker^™^ Green FM was read in the Fluorescein Isothiocyanate Channel (Excitation: 498 nm; Emission: 517 nm) on a BD Fortessa flow cytometer (BD Biosciences, San Jose, CA, USA) with gating parameters set up using the BD FACSDiva software (Rickard et al., 2025).

#### 2.6.2. Evaluation of superoxide levels

Following the timeline and plating procedure used for TMRE flow cytometry, cells were harvested and stained with MitoSOX^™^ dye (Invitrogen^™^, for superoxide detection). 2.5 μL of a 1 mM MitoSOX^™^ dye stock (5 μM final concentration) was incubated with cells for 30 min at 37^◦^C. MitoSOX^™^ fluorescence was read using the PE-Texas Red Channel (Excitation: 561 nm; Emission: 610 nm) on a BD Fortessa flow cytometer with gating parameters set up using the BD FACSDiva software.

#### 2.6.3. Evaluation of mtDNA copy number

Outgrown and re-exposed OVCAR-3 cells were seeded in 12-well plates at 1.6 * 10^4^ cells/cm^2^ prior to exposure to complete medium or PFAS following the dosing scheme above. On day 8, the medium was removed, and cells were detached from the plate as previously described. Genomic DNA was isolated using the QIAamp DNA Mini Kit (Qiagen, Hilden, Germany) following manufacturer’s instructions and stored at −20°C until analysis. Following published methods (Rickard et al., 2025), RT-qPCR was used to measure hMT-ND1, a mitochondrial gene, and h18S-RNA with a CFX384 Real-Time System C1000 Touch Thermal Cycler (Bio-Rad Laboratories, Hercules, CA, USA) using the recommended cycling parameters for SYBR^®^ Green Supermix. Primer efficiencies for hMT-ND1 and h18S-RNA were 2.12 and 2.3, respectively, which were used to compute fold changes in ND1 and 18S-RNA copy numbers.

### 2.7. Statistical analyses

**Fig. 1.**
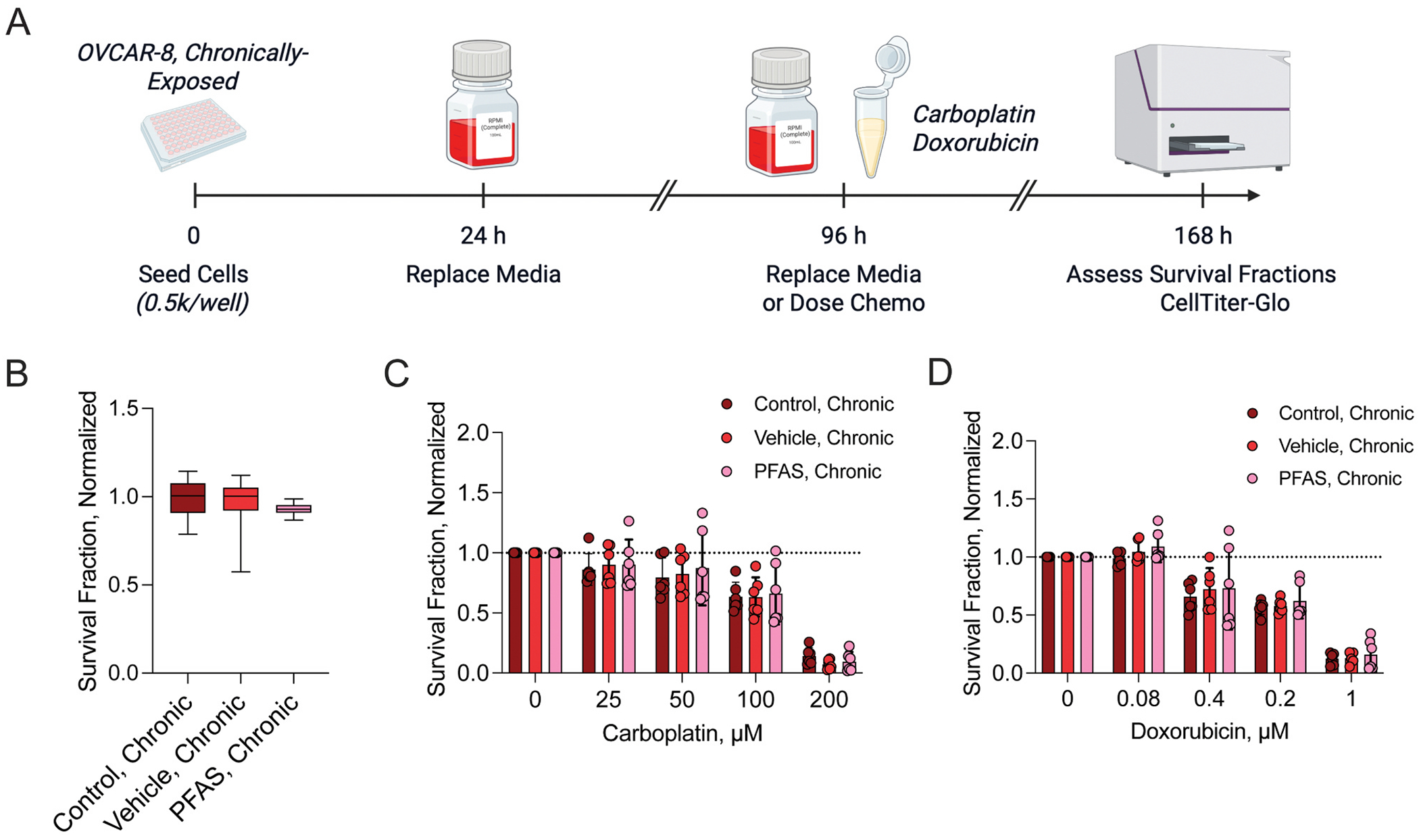
Effects of chronic PFAS exposure on OVCAR-8 cell survival and chemotherapy response. A) Timeline of experiments. Survival fraction of (B) OVCAR-8 PFAS chronically-exposed cells compared to vehicle-exposed and unexposed cells, (C) OVCAR-8 chronically-exposed cells following carboplatin treatment, and (D) OVCAR-8 chronically-exposed cells following doxorubicin treatment. Data represent n = at least 3 biological replicates with 2 technical replicates and are normalized to the respective control. Dashed line represents survival fraction of respective control groups. Timeline created in BioRender.

**Fig. 2.**
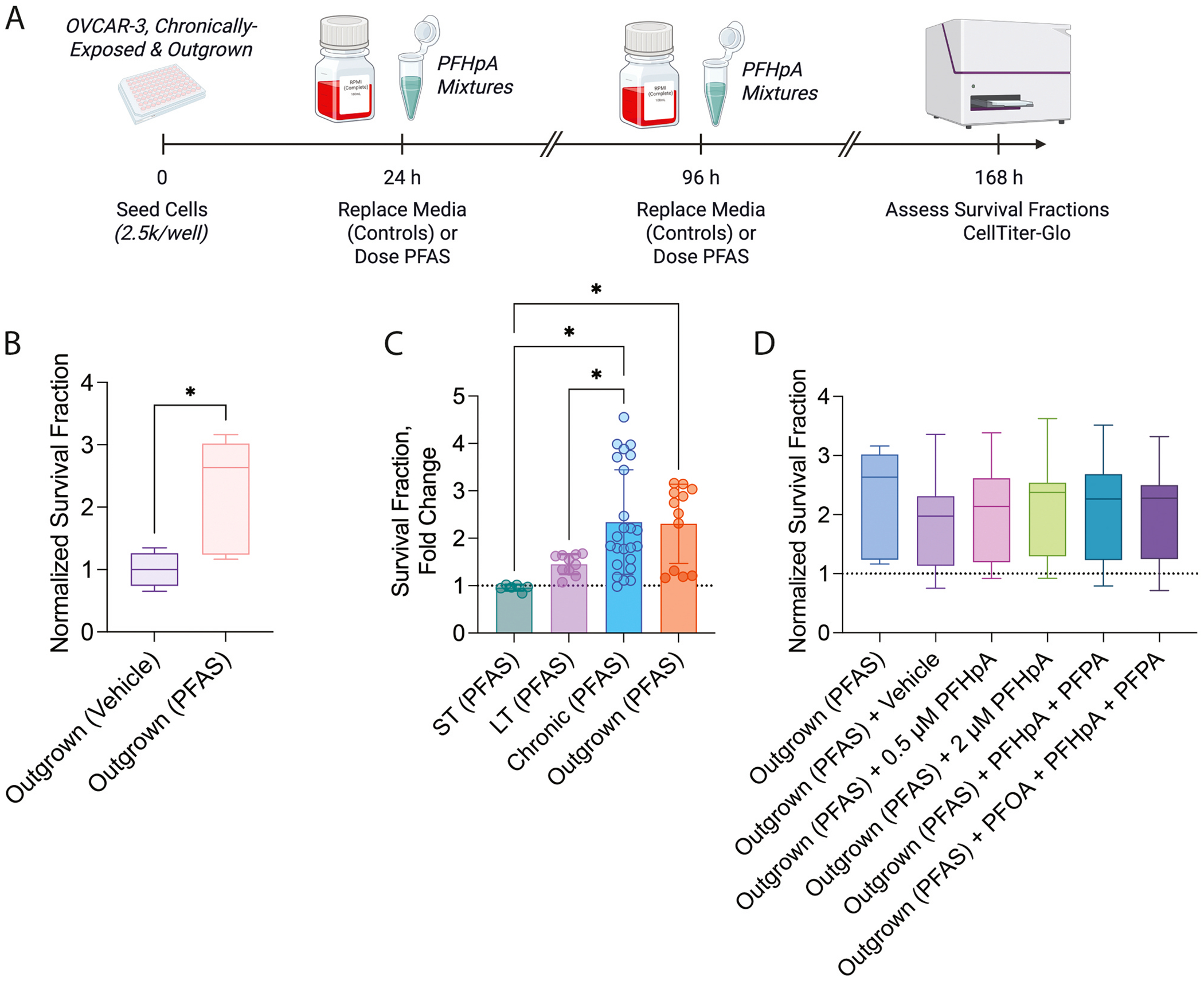
Effects of recovery or re-exposure on chronically-exposed OVCAR-3 cells. A) Timeline of experiments. Survival fraction of (B) outgrown PFAS-exposed cells compared to outgrown vehicle-exposed OVCAR-3 cells, (C) outgrown PFAS-exposed cells compared to short-term (ST, 48 h), long-term (LT, 6 d), and PFAS chronically-exposed cells (26–35 d), or (D) outgrown cells re-exposed to vehicle, PFHpA, or PFAS mixtures. Graph (C) depicts several previously published data (Rickard et al. 2025). Data represent n = at least 3 biological replicates with 2 technical replicates and are normalized to the respective control (dashed line). Dashed line represents survival fraction of respective control groups. Significant differences between PFAS exposure group versus respective control determined by unpaired *t*-test or one-way ANOVA and denoted by * (*p* < 0.05). Timeline created in BioRender.

**Fig. 3.**
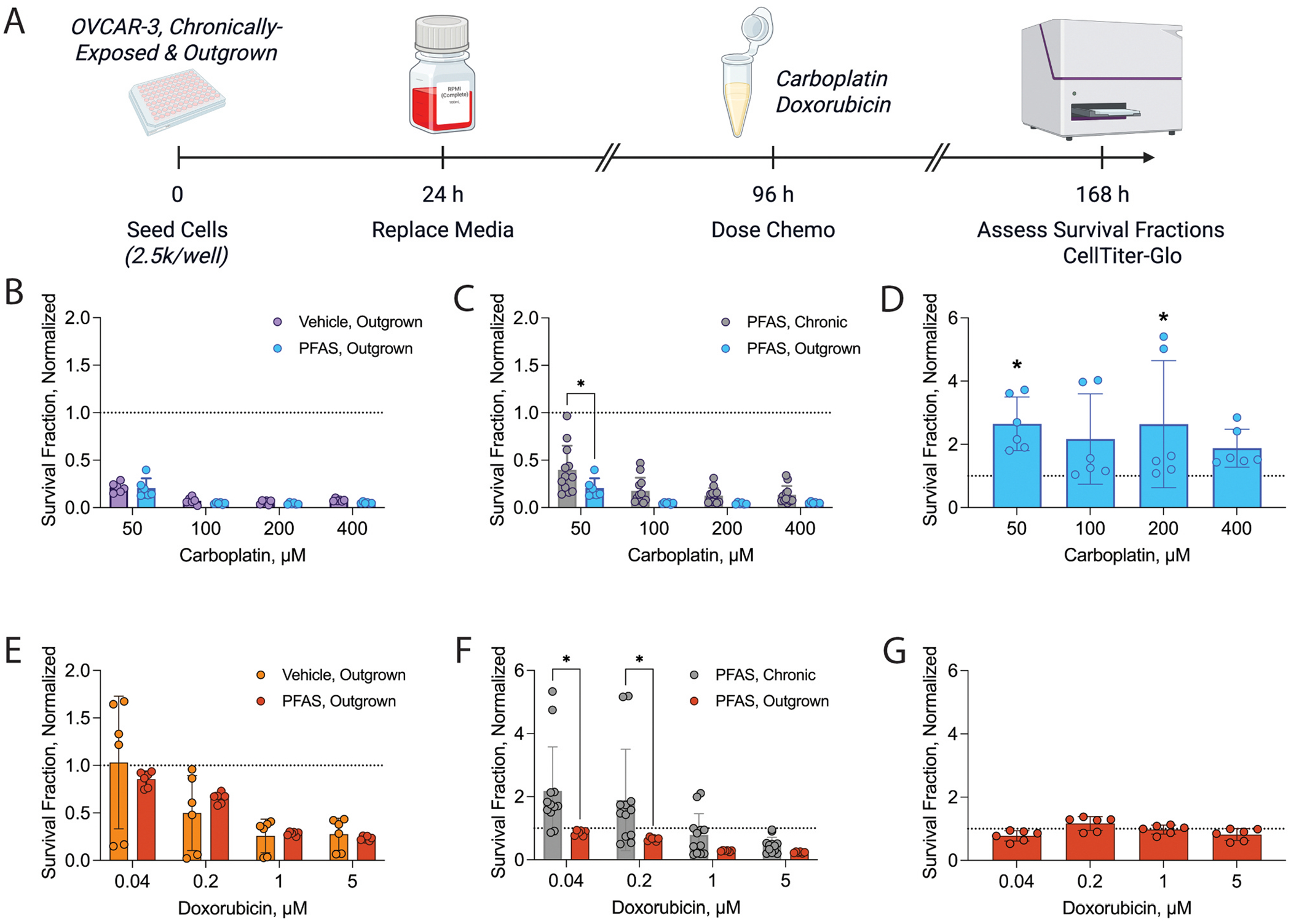
Effects of recovery on survival fraction in OVCAR-3 cells post-chemotherapy treatment. A) Timeline of experiments. Survival fraction of outgrown cells normalized to the respective control or PFAS chronically-exposed cells following treatment with (B, C) carboplatin or (E, F) doxorubicin. Alternate visualizations of data: comparison of survival fraction between outgrown cells and controls when data are normalized to vehicle group at each respective (D) carboplatin or (G) doxorubicin concentration. Previously published data shown as grey bars (Rickard et al. 2025). Data represent n = at least 3 biological replicates with 2 technical replicates. Dashed line represents survival fraction of respective control groups. Significant differences between PFAS exposure group versus respective control determined by two-way ANOVA with Šídák’s multiple comparisons test or unpaired *t*-tests and denoted by * (*p* < 0.05). Timeline created in BioRender.

**Fig. 4.**
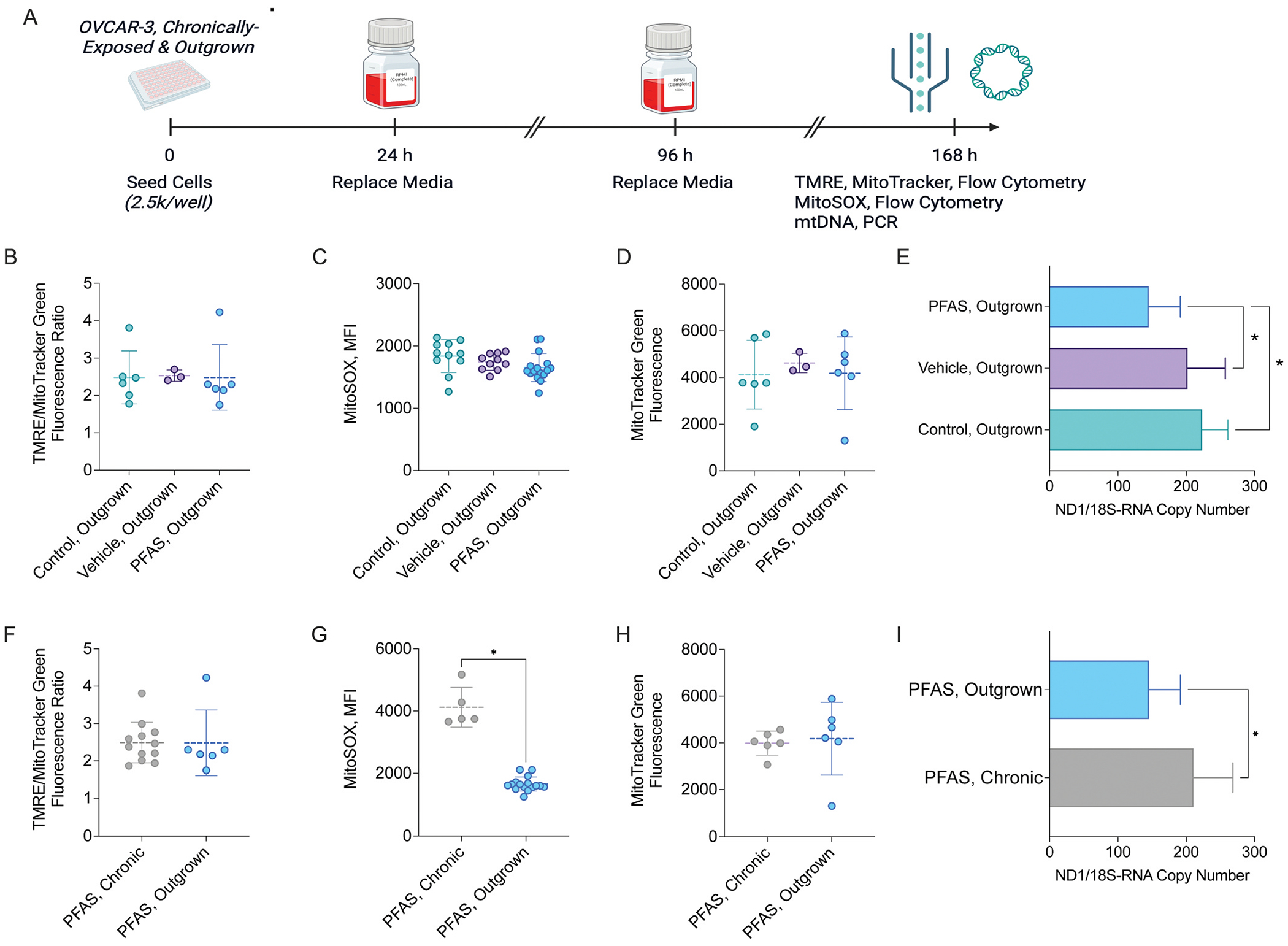
Effects of recovery on mitochondrial membrane potential, superoxide, content, and mtDNA copy number in PFAS chronically-exposed OVCAR-3 cells. A) Timeline of experiments. Comparison of (B, F) TMRE to MitoTracker^™^ Green MFI ratio, (C,G) MitoSOX^™^ MFI, or (D, H) MitoTracker^™^ Green MFI between all outgrown cell types or outgrown vs. PFAS chronically-exposed cells. Comparison of ND1–18S-RNA copy number ratio between (E) all outgrown cell types or (I) outgrown vs. PFAS chronically-exposed cells. Data represent n = at least 3 biological replicates with 1 pooled technical replicate. Significant differences between PFAS outgrown versus PFAS chronically-exposed group determined by unpaired *t*-tests and denoted by * (*p* < 0.05). Timeline created in BioRender.

To evaluate the effect of PFAS outgrowth (recovery) or re-exposure on outcomes of interest (survival fraction, ΔΨ_m_, superoxide, mitochondrial content, and mtDNA copy number), unpaired *t*-tests, one-way ANOVA, or two-way ANOVA were used as appropriate (unpaired *t*-test: Figs. 2B, 3D and G, 4F–I, S3A; one-way ANOVA: Figs. 1B, 2C–D, 4B–E, S3B–C, S4, S5; two-way ANOVA: Figs. 1C–D, 3B–C and E–F, S1, S2). All tests were 2-sided at alpha level 0.05 unless otherwise specified. All analyses were performed in Prism 10.0 software (GraphPad, San Diego, CA, USA).

## 3. Results

Firstly, we sought to determine the effects of chronic PFAS exposure in chemoresistant OVCAR-8 cells (Fig. 1A). Unlike OVCAR-3 cells, OVCAR-8 cell survival fraction, as measured by adenosine triphosphate (ATP) content, was unaffected by chronic vehicle or PFAS exposure (Fig. 1B). Additionally, OVCAR-8 cell response to neither carboplatin nor doxorubicin was altered by chronic PFAS exposure (Fig. 1C–D), suggesting minimal PFAS-induced effects in this cell line.

Since several survival advantages were observed in OVCAR-3, but not OVCAR-8, cells following chronic PFAS exposure, we next examined whether these endpoints could revert to baseline following “recovery” from PFAS exposure (“outgrown”; Fig. 2A). We hypothesized that allowing cells to recover from PFAS exposure would overcome mitochondria-related alterations and re-sensitize ovarian cancer cells to chemotherapy. Compared to vehicle-exposed cells (1 ± 0.3), the survival fraction of outgrown PFAS-exposed OVCAR-3 cells remained significantly elevated (2.3 ± 0.8, p < 0.001; Fig. 2B); in fact, survival fraction was not significantly different than that of LT or chronic PFAS exposures, both of which displayed increases compared to ST exposures (Fig. 2C). When outgrown cells were re-exposed to PFAS for 6 d, no significant changes in survival fraction were observed above the already elevated level (Fig. 2D). These findings suggest that effects of chronic PFAS exposure on cell proliferation were not reversed following recovery.

Next, we wanted to identify whether chemotherapy response improved in the absence of chronic PFAS exposure (Fig. 3A). Contrary to PFAS chronically-exposed cells, where carboplatin and doxorubicin resistance were observed at each concentration tested (Rickard et al., 2025), outgrown cells responded better to chemotherapy treatment. No significant increases in survival fraction post-carboplatin (Fig. 3B) or doxorubicin (Fig. 3E) treatment were observed when normalized to respective no treatment controls. In fact, compared to chronically-exposed cells, significant decreases in survival fraction were observed in outgrown PFAS-exposed cells treated with 50 μM carboplatin (outgrown: 0.2 ± 0.1, chronically-exposed: 0.4 ± 0.3; p < 0.05) and 0.04 (outgrown: 0.9 ± 0.1, chronically-exposed: 2.2 ± 1.4, p < 0.05), or 0.2 μM doxorubicin (outgrown: 0.7 ± 0.1, chronically-exposed: 1.9 ± 1.6, p < 0.05) (Fig. 3C, F). When survival fraction of outgrown PFAS-exposed cells was normalized to outgrown vehicle-exposed cells at each respective chemotherapy dose, significant increases were observed in cells treated with 50 μM (2.7 ± 0.9, p < 0.05) or 200 μM carboplatin (2.6 ± 2, p < 0.05; Fig. 3D), but not doxorubicin (Fig. 3G). While resistance was observed at 50 μM, the significant variability observed at 200 μM could suggest that resistance only occurs when treated with low-dose carboplatin. Importantly, survival fraction post-chemotherapy treatment was unaffected in outgrown vehicle-exposed cells compared to unexposed cells, indicating no effect of the vehicle itself (Fig. S1). These findings suggest that recovery from chronic PFAS exposure can partially or fully re-sensitize OVCAR-3 cells to carboplatin or doxorubicin treatment, respectively.

Because recovery from chronic PFAS exposure re-sensitized cells to select chemotherapy concentrations, we then tested how re-exposure of outgrown cells to PFAS affected chemotherapy response (Fig. S2A). When survival fraction was normalized to the no treatment control for each re-exposure group, several instances of significantly altered survival fractions were observed following treatment with 50 μM carboplatin (Fig. S2B) or 0.04-1 μM doxorubicin (Fig. S2D), mainly in groups that were exposed to higher concentrations of PFAS or PFAS mixtures. When survival fraction of re-exposed cells was normalized to the outgrown group at each respective chemotherapy concentration, no significant changes were observed in cells treated with carboplatin (Fig. S2C) or doxorubicin (Fig. S2E). These findings indicate that outgrown cells may have increased chemotherapy sensitivity upon reexposure to higher PFAS concentrations or PFAS mixtures, both of which have been previously shown to be more toxic than low dose PFAS (Pierozan et al., 2023).

To understand mechanisms driving the enhanced chemosensitivity of outgrown cells compared to chronically-exposed cells, we next examined mitochondrial functional parameters (Fig. 4A). When measuring ΔΨ_m_ using the JC-1 assay (no mitochondrial content normalization), significantly decreased ΔΨ_m_ was observed in PFAS-exposed outgrown cells compared to PFAS chronically-exposed cells (Fig. S3A). This suggests the return of ΔΨ_m_ to near baseline levels, since ΔΨ_m_ was still slightly elevated in PFAS outgrown cells compared to controls (Fig. S3B) and could indicate increased mitochondrial activity. Conversely, when outgrown cells were re-exposed to PFAS, significantly decreased ΔΨ_m_ was observed in the PFHpA + PFPA group compared to vehicle-exposed cells, possibly indicating mixture-induced toxicity (Fig. S3C). When ΔΨ_m_ was measured via TMRE and normalized to mitochondrial content, no changes were observed in outgrown cells of any type (Fig. 4B) or even following re-exposure to PFAS compared to controls (Fig. S4B); however, this was expected as even PFAS chronically-exposed cells did not display altered ΔΨ_m_ when evaluated using this method (Fig. 4F). Since the use of TMRE normalized to MitoTracker^™^ is considered a more sensitive readout of ΔΨ_m_, we conclude that ΔΨ_m_ is largely unaffected by chronic PFAS exposure.

Although ΔΨ_m_ was unchanged, long duration PFAS exposure can affect mitochondrial energy pathways and ROS production (Rickard et al., 2025). Thus, we compared superoxide levels, as a proxy of electron transport chain activity, in PFAS chronically-exposed cells and outgrown cells. In chronically-exposed OVCAR-3 cells, MitoSOX^™^ mean fluorescence intensity (MFI) increased in both the vehicle- (5246 ± 787) and PFAS-exposed (4121 ± 637) groups compared to unexposed cells (2477 ± 495, p < 0.04; Fig. S5), suggesting an adaptive stress response to toxicant exposure. In outgrown cells, no alterations in MitoSOX^™^ MFI were observed between controls and PFAS-exposed groups (Fig. 4C). MitoSOX^™^ MFI was significantly decreased in PFAS outgrown cells (1655 ± 226) compared to PFAS chronically-exposed cells (Fig. 4G; 4121 ± 637, p < 0.001); however, it is important to note that vehicle chronically-exposed cells also displayed increased MitoSOX^™^ MFI. MitoSOX^™^ MFI was unaffected when outgrown cells were re-exposed to PFAS (Fig. S4C).

Since ΔΨ_m_ was largely unaffected by recovery from PFAS exposure when normalized to mitochondrial content, we hypothesized that mitochondrial content, which increased in PFAS chronically-exposed cells (Rickard et al., 2025), would be reduced in outgrown cells. When mitochondrial content was measured via MitoTracker^™^ Green, no significant changes were observed in outgrown PFAS-exposed cells compared to controls or PFAS chronically-exposed cells (Fig. 4D, H), however much more variability was noted in outgrown cell groups. MitoTracker^™^ Green MFI was also unchanged in all groups following re-exposure when normalized to the vehicle group (Fig. S4D).

As a secondary measurement for mitochondrial content, mtDNA copy number was evaluated. Compared to unexposed (223 ± 38) and vehicle controls (202 ± 55), mtDNA copy number significantly decreased in PFAS outgrown cells (Fig. 4E, 145 ± 46, p < 0.01). Additionally, compared to PFAS chronically-exposed cells, which demonstrated significantly increased mtDNA copy numbers compared to their respective controls (200 ± 56), mtDNA copy number significantly decreased in outgrown cells (p < 0.001; Fig. 4I). When outgrown cells were re-exposed to vehicle or PFAS, trends towards increased mtDNA copy number were observed in all groups including vehicle-exposed cells; however, the only significant increase occurred in the 0.5 μM PFHpA group (Fig. S4E).

## 4. Discussion

In the present study, we evaluated the potential for chronic PFAS exposure to affect survival fraction and chemotherapy response in a chemoresistant, molecularly and bioenergetically distinct ovarian cancer cell line, OVCAR-8. Since no significant changes in survival or chemotherapy response were observed, this suggests that the effects of PFAS on each cell line, even those representing similar cancer types, are unique. Additionally, these findings could indicate that ovarian cancer cells with higher chemosensitivities are more susceptible to developing resistance following environmental exposures and warrant further investigation.

It is estimated that as many as 200 million Americans are living in a community with drinking water supplies contaminated by PFAS (Andrews and Naidenko, 2020). As a result, those affected may make lifestyle changes to reduce exposure. While removing PFAS entirely is not human-relevant, allowing OVCAR-3 cells to “recover” from PFAS exposure proved effective at reversing several PFAS-induced effects. For example, only limited instances of chemoresistance, restricted to carboplatin, persisted in outgrown cells.

When evaluating mitochondria-related alterations, outgrown cells displayed no change in ΔΨ_m_ when normalized to mitochondrial content. The lack of significant alterations may be due to the transient nature of ΔΨ_m_ (flow cytometry requires cell harvesting, while other assays can be readout immediately) or PFAS-related mitochondrial content alterations. Despite minimal changes in ΔΨ_m_, the observed decrease in MitoSOX^™^ MFI in outgrown cells suggests that recovery from chronic vehicle or PFAS exposure allows mitochondrial superoxide levels to decrease, potentially through a decrease in electron transport chain activity.

Similar to ΔΨ_m_, outgrown cells displayed no change in mitochondrial content when measured via MitoTracker^™^ but significantly decreased mtDNA copy number compared to chronically-exposed cells. This discrepancy may be due to increased precision of PCR for measuring this endpoint or methodological differences between protocols. For instance, for PCR, cells were pooled from two wells which may increase the representative cell population and enable more sensitive detection of changes. Nonetheless, this decrease in mtDNA copy number may be associated with the observed improvement in chemotherapy response. While studies have reported both increased and decreased mtDNA copy number to be associated with malignant progression of ovarian cancer (Meng et al., 2019; Shukla and Singh, 2021; Wang et al., 2006), others have reported that, in different tumor types, decreases in mtDNA copy number are often accompanied by ROS generation, chemosensitivity, and apoptosis (Mei et al., 2015).

Despite modest reversal of mtDNA alterations and chemoresistance, survival fraction in outgrown cells remained significantly elevated after recovery. This suggests that the mechanisms driving PFAS-induced proliferation require longer recovery durations, differ from those regulating mitochondrial changes and chemoresistance, or that chronic PFAS exposure reprograms the cell in such a way that increased proliferation is sustained even after the removal of exposure.

While measures can be taken to decrease daily PFAS exposures, other actions, like traveling to a new geographic area, can introduce new PFAS exposures. To mimic this potential increase in exposure, outgrown cells were re-exposed to PFAS or PFAS-containing mixtures. While survival was unaffected after re-exposure, increased variability was noted in all groups, which may result from instability induced by increased durations of PFAS exposure or PFAS-related alterations in proliferation pathways.

Compared to outgrown cells, re-exposed groups also displayed significant increases in mitochondrial content measured via MitoTracker^™^ Green. mtDNA copy number trended towards an increase in most groups as well but was only statistically significant for 0.5 μM PFHpA. Since mtDNA copy number trended towards an increase even following vehicle exposure, this may indicate an adaptive stress response to secondary toxic exposures and warrants further investigation. Observed increases in mitochondrial content could result from mitochondrial biogenesis, which has been linked with increased bioenergetics in ovarian cancer (Koc et al., 2023; Shen and Zhan, 2022). Altered bioenergetics, or metabolic reprogramming, a hallmark of cancer, is known to play a role in cell growth and the development of chemoresistant cell populations (Dar et al., 2017; Hanahan and Weinberg, 2011). Thus, future studies should evaluate bioenergetics in ovarian cancer cells that have endured chronic PFAS exposure to better understand pathways driving proliferative and mitochondrial effects.

To our knowledge, this is the first study exploring the ability of an *in vitro* model to recover from chronic PFAS exposure. While this study presents some potential benefits of a “recovery” period from PFAS exposure on ovarian cancer cells, it is not yet understood how this translates clinically or affects other cell types within the tumor micro-environment and other bodily systems. Additionally, although PFAS have been detected in follicular fluid, suggesting their accumulation in the female reproductive tract, their distribution to ovarian tissue has not been measured. Thus, future work should measure ovarian PFAS levels in *in vivo* models or patient tissue samples to better understand potential ovary-specific benefits of PFAS recovery. If *in vivo* models recapitulate chemoresistance, precise mechanistic drivers or biomarkers associated with altered therapy response could be identified. *In vivo* models would also enable evaluation of multi-system effects of chronic PFAS exposure and recovery, which could inform follow-up studies or clinical populations of interest. Finally, since recovery from PFAS exposure may prove effective at reversing adverse cellular outcomes, epidemiologic studies measuring PFAS exposures would benefit from including information on factors influencing PFAS exposure levels, such as the use of water filtration systems, conscious phasing out of PFAS-containing consumer goods, or recent geographical re-location.

## Supplementary Material

1

## Data Availability

A publicly available link containing all data will be provided upon request.

## Appendix A. Supporting information

Supplementary data associated with this article can be found in the online version at doi:10.1016/j.toxlet.2026.111858.

## Abbreviations:

ΔΨ_m_: mitochondrial membrane potential
ATP: adenosine triphosphate
D: days
DMSO: dimethyl sulfoxide
FBS: fetal bovine serum
H: hours
HEPES: 2-[4-(2-hydroxyethyl)piperazin-1-yl]ethanesulfonic acid
JC-1: 5,5′,6,6′,-tetrachloro-1,1′,3,3′-tetraethylbenzimidazolylcarbocyanine iodide
LT: long-term
MFI: mean fluorescence intensity
mtDNA: mitochondrial deoxyribonucleic acid
PBS: phosphate buffered saline
PFAS: per- and polyfluoroalkyl substances
PFHpA: perfluoroheptanoic acid
PFOA: perfluorooctanoic acid
PFPA: perfluoropentanoic acid
ROS: reactive oxygen species
ST: short-term
TMRE: tetramethylrhodamine ethyl ester
