# Supplementary material for: Recovery from chronic PFAS exposure can reverse chemotherapy resistance and mitochondrial alterations in ovarian cancer cells
Source: Toxicol Lett. Author manuscript; Available in PMC 2026 Jun 3. (PMC13231427; doi:10.1016/j.toxlet.2026.111858)
Supplement: 1 [file NIHMS2178805-supplement-1.docx]

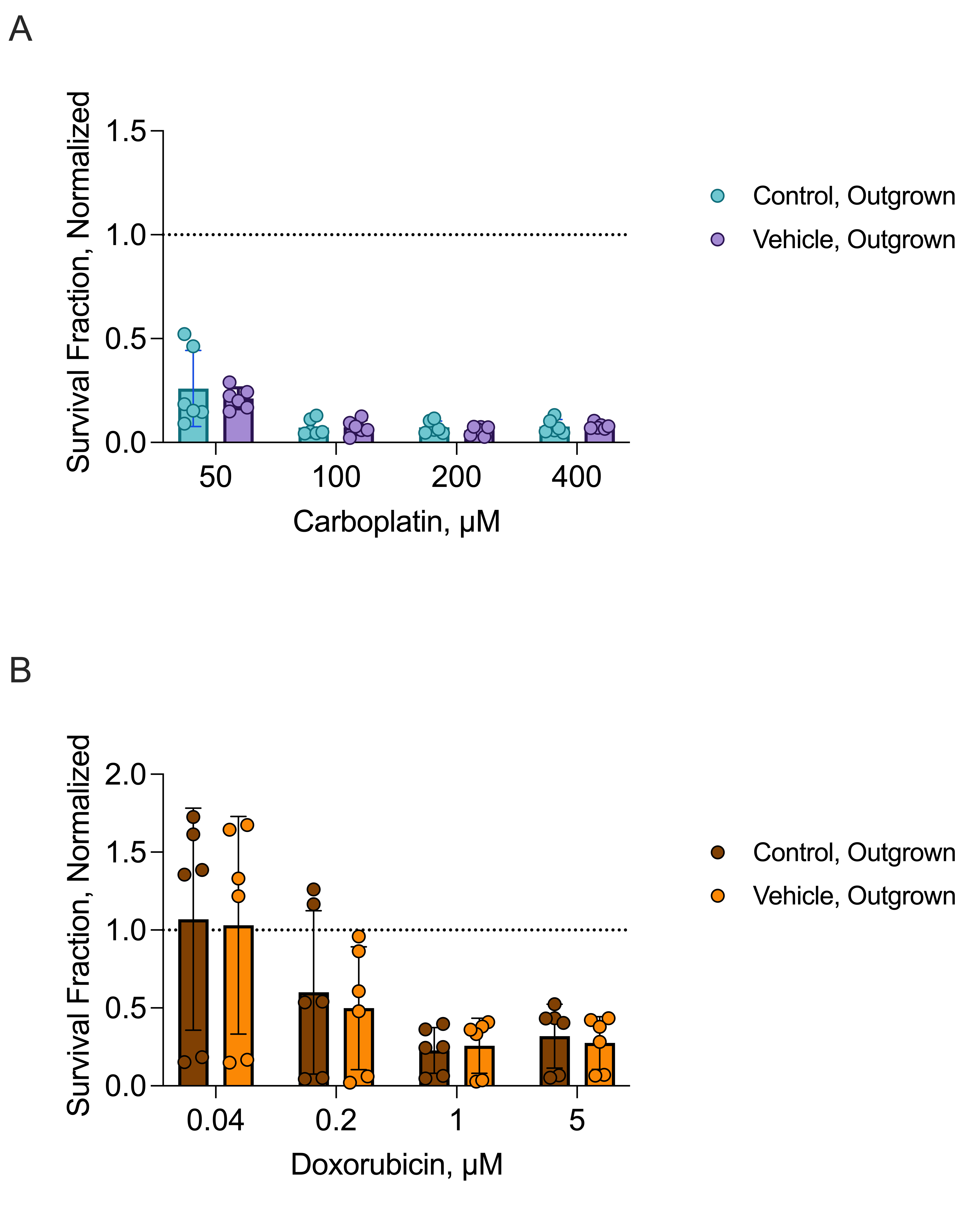


**Figure S1. Effects of vehicle on outgrown OVCAR-3 cell response to chemotherapy.** Survival fraction of control OVCAR-3 outgrown versus vehicle OVCAR-3 outgrown cells following A) carboplatin or B) doxorubicin treatment. Data represent n=3 biological replicates with 2 technical replicates each and are normalized to the respective control (dashed line).


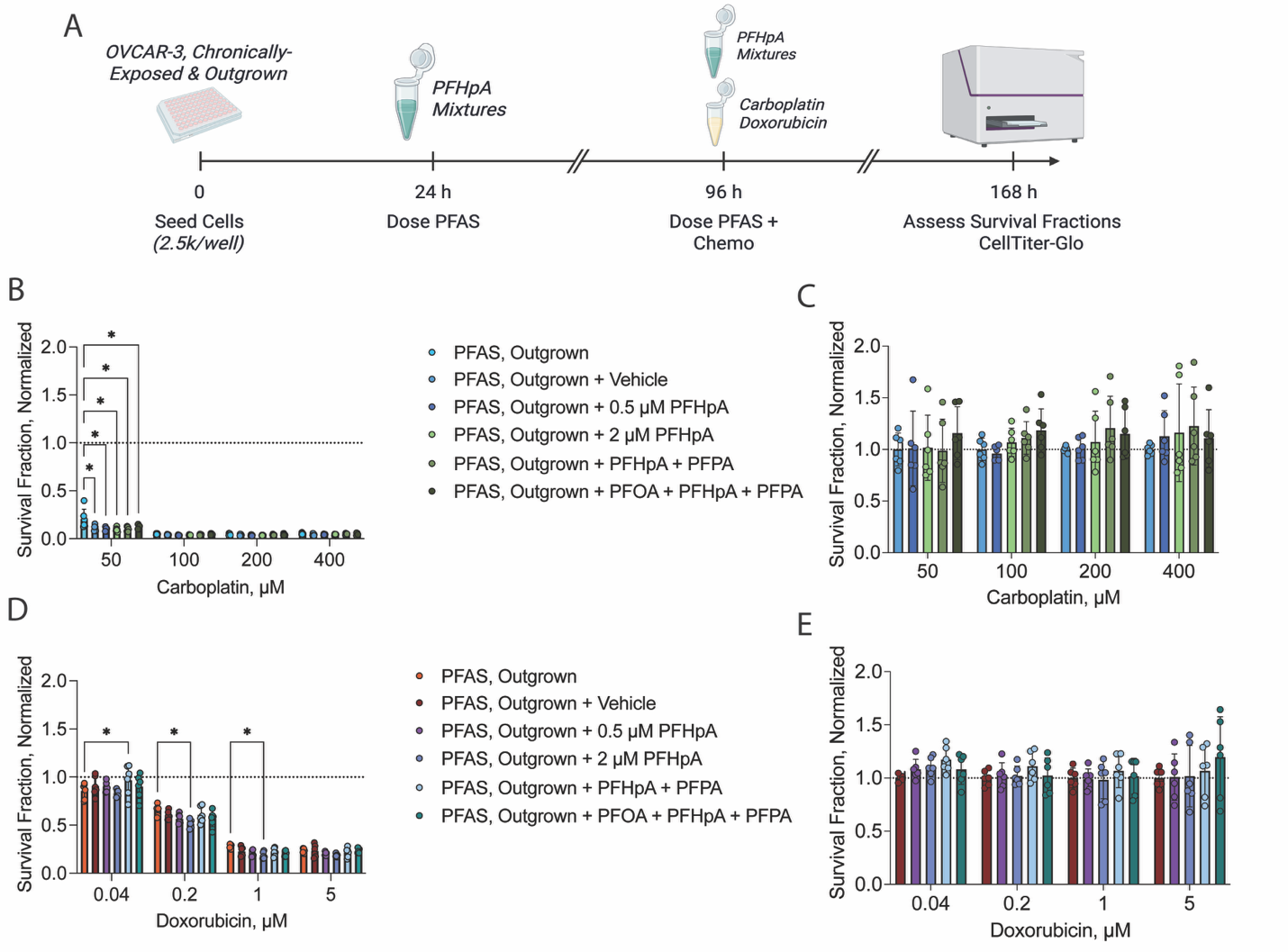


**Figure S2. Effects of PFAS re-exposure on the survival fraction of outgrown PFAS chronically-exposed OVCAR-3 cells post-chemotherapy treatment.** A) Timeline of experiments. B) Survival fraction of outgrown PFAS chronically-exposed cells compared to outgrown PFAS chronically-exposed cells that were re-exposed to vehicle, PFHpA, or PFAS mixtures and treated with carboplatin. C) Alternate visualization of data in B); Comparison of survival fraction between outgrown PFAS chronically-exposed OVCAR-3 cells and re-exposed cells when data are normalized to the re-exposed vehicle group at each respective carboplatin concentration. D) Survival fraction of outgrown PFAS chronically-exposed cells compared to outgrown PFAS chronically-exposed cells that were re-exposed to vehicle, PFHpA, or PFAS mixtures and treated with doxorubicin. E) Alternate visualization of data in D); Comparison of survival fraction between outgrown PFAS chronically-exposed OVCAR-3 cells and re-exposed cells when data are normalized to the re-exposed vehicle group at each respective doxorubicin concentration. Data represent n= 3 biological replicates with 2 technical replicates each and are normalized to the respective control (PFAS, outgrown group in B) and D), respective vehicle control at each chemotherapy concentration in C) and E)). Dashed line represents survival fraction of respective control groups. Significant differences between re-exposure group versus respective control determined by two-way ANOVA with Dunnett’s multiple comparisons test and denoted by * (*p* < 0.05). Timeline created in BioRender.


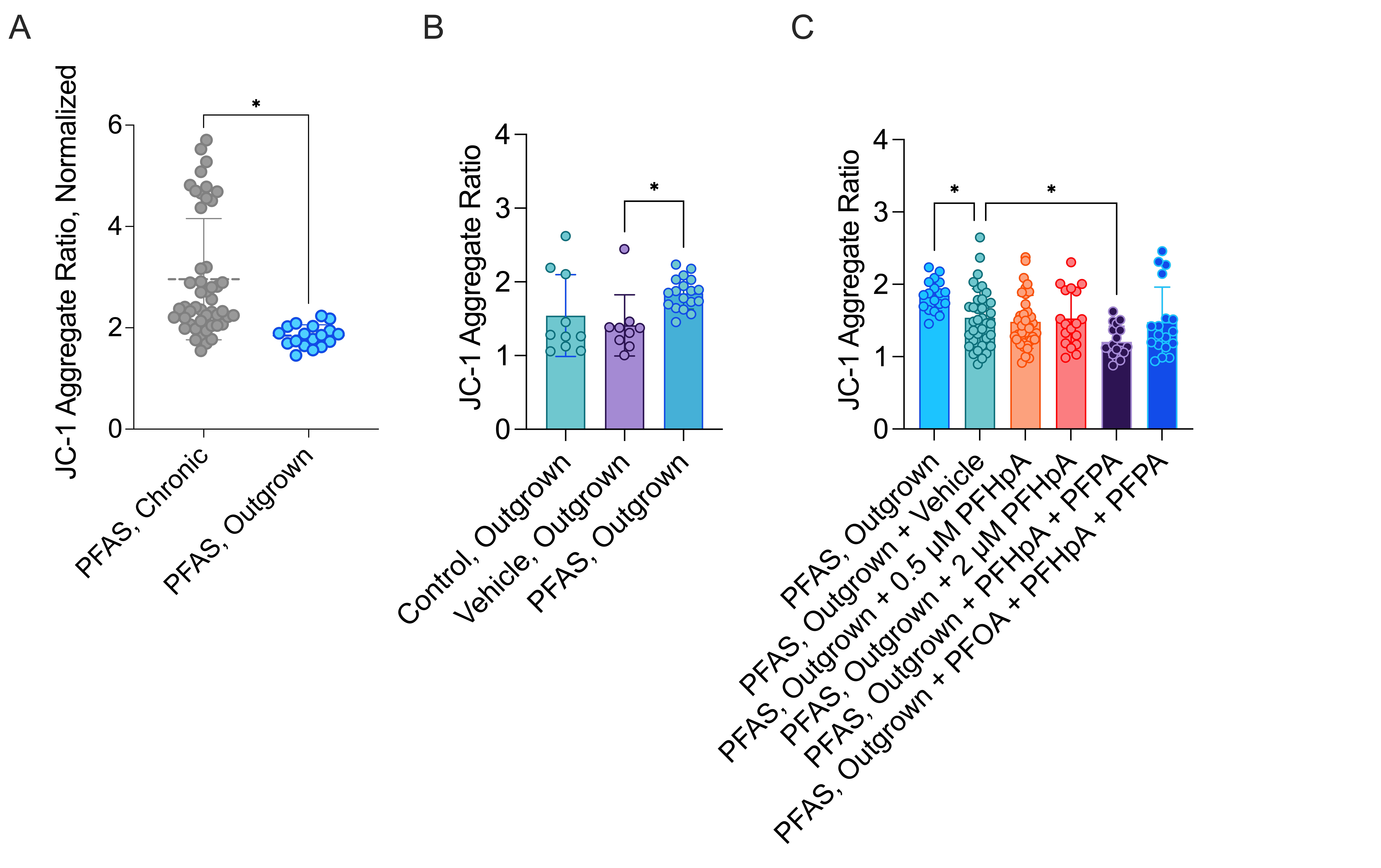


**Figure S3. Effects of recovery (outgrowth) or re-exposure on OVCAR-3 mitochondrial membrane potential.** Comparison of JC-1 aggregate ratio in (A) outgrown PFAS chronically-exposed cells versus PFAS chronically-exposed OVCAR-3 cells, (B) outgrown control, vehicle, and PFAS chronically-exposed OVCAR-3 cells, and (C) outgrown PFAS chronically-exposed cells versus re-exposed outgrown OVCAR-3 cells. Data represent n= at least 3 biological replicates with 2 technical replicates each and are normalized to the respective control. Significant differences between outgrown PFAS chronically-exposed cells versus outgrown vehicle chronically-exposed cells or re-exposure groups determined by unpaired t-test or one-way ANOVA and denoted by * (*p* < 0.05).


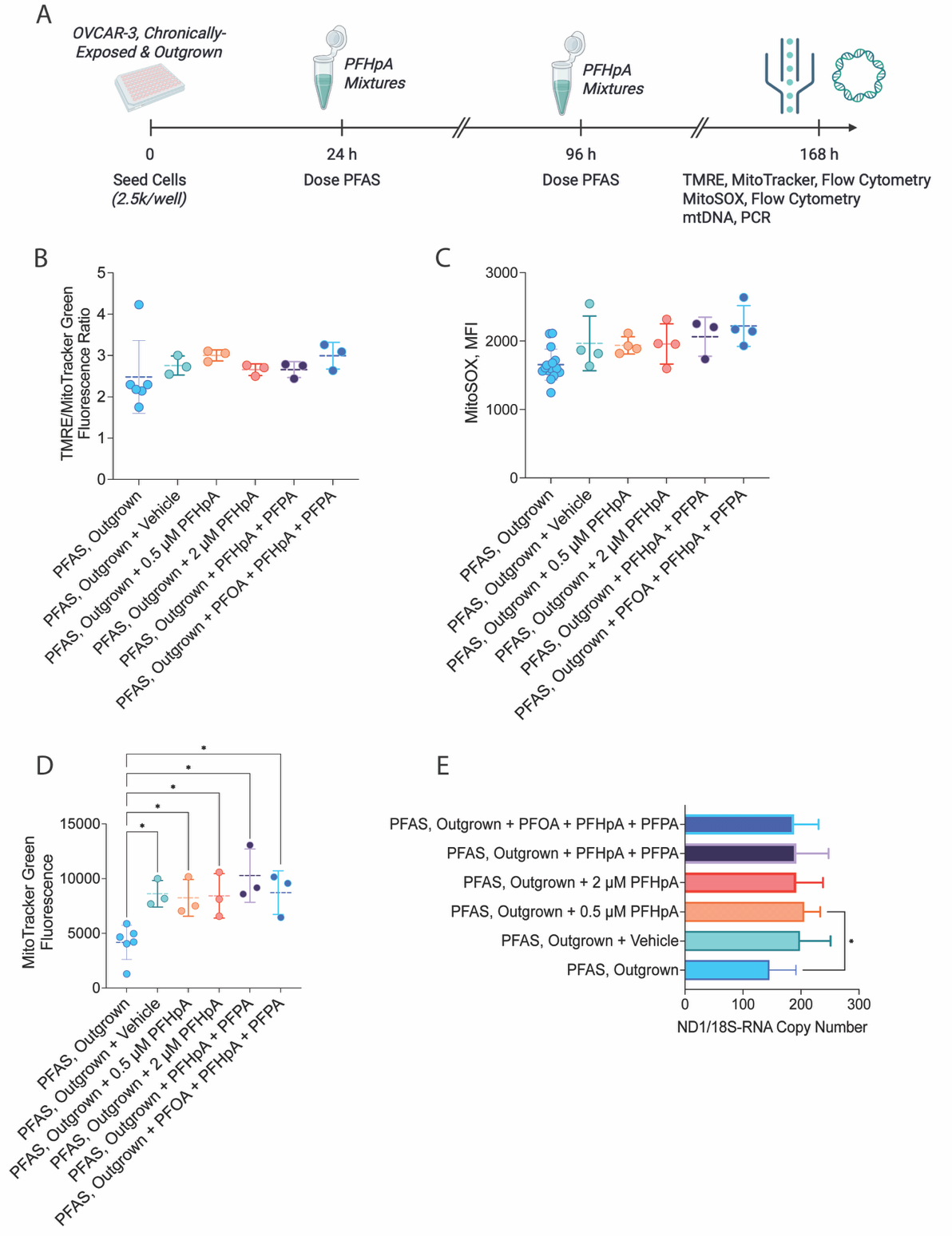


**Figure S4. Effects of PFAS re-exposure on mitochondrial membrane potential, superoxide production, content, and mtDNA copy number in outgrown PFAS chronically-exposed OVCAR-3 cells.** A) Timeline of experiments. Comparison of TMRE to MitoTracker™ Green MFI ratio (B), MitoSOX MFI (C), MitoTracker Green MFI (D), and ND1 to 18S-RNA copy number ratio between PFAS outgrown OVCAR-3 cells and those re-exposed to vehicle, PFHpA, and PFAS mixtures. Data represent n= at least 3 biological replicates with 1 pooled technical replicate each. Significant differences between PFAS outgrown versus PFAS re-exposed group determined by one-way ANOVA and denoted by * (*p* < 0.05). Timeline created in BioRender.

**Figure S5. Effects of chronic exposures on MitoSOX™ levels in OVCAR-3 cells.** Comparison of MitoSOX MFI between unexposed, vehicle-, and PFAS-chronically exposed OVCAR-3 cells. Data represent n= 3 biological replicates with at least 1 technical replicate each. Significant differences between PFAS group and controls determined by one-way ANOVA and denoted by * (*p* < 0.05).
